# Leveraging Wearable Sensors for Human Daily Activity Recognition with Stacked Denoising Autoencoders

**DOI:** 10.3390/s20185114

**Published:** 2020-09-08

**Authors:** Qin Ni, Zhuo Fan, Lei Zhang, Chris D. Nugent, Ian Cleland, Yuping Zhang, Nan Zhou

**Affiliations:** 1 College of Information, Mechanical and Electrical Engineering, Shanghai Normal University, Shanghai 201418, China; niqin@shnu.edu.cn (Q.N.); 1000459457@smail.shnu.edu.cn (Z.F.); yp_zhang@shnu.edu.cn (Y.Z.); zn1016807290@outlook.com (N.Z.); 2 College of Information Science and Technology, Donghua University, Shanghai 201620, China; 3 School of Computing and Mathematics, University of Ulster, Belfast BT370QB, UK; cd.nugent@ulster.ac.uk (C.D.N.); i.cleland@ulster.ac.uk (I.C.)

**Keywords:** activity recognition, transitional activities, stacked denoising autoencoders, wearable sensors, resampling technique

## Abstract

Activity recognition has received considerable attention in many research fields, such as industrial and healthcare fields. However, many researches about activity recognition have focused on static activities and dynamic activities in current literature, while, the transitional activities, such as stand-to-sit and sit-to-stand, are more difficult to recognize than both of them. Consider that it may be important in real applications. Thus, a novel framework is proposed in this paper to recognize static activities, dynamic activities, and transitional activities by utilizing stacked denoising autoencoders (SDAE), which is able to extract features automatically as a deep learning model rather than utilize manual features extracted by conventional machine learning methods. Moreover, the resampling technique (random oversampling) is used to improve problem of unbalanced samples due to relatively short duration characteristic of transitional activity. The experiment protocol is designed to collect twelve daily activities (three types) by using wearable sensors from 10 adults in smart lab of Ulster University, the experiment results show the significant performance on transitional activity recognition and achieve the overall accuracy of 94.88% on three types of activities. The results obtained by comparing with other methods and performances on other three public datasets verify the feasibility and priority of our framework. This paper also explores the effect of multiple sensors (accelerometer and gyroscope) to determine the optimal combination for activity recognition.

## 1. Introduction

Activity recognition, as an important application, has been received considerable attention and is widely used in many research fields, such as industrial and healthcare fields [1]. In general, activity recognition means that people’s daily behavior types are identified by a large amount of human behavior information that is collected by a variety of channels which include camera [2], microphone [3], sensor [4,5], and so on. Moreover, for activity recognition, research has found four dominant groups of technologies and two emerging technologies: smartphones, wearables, video, electronic components, Wi-Fi, and assistive robots [6]. Wearable sensors that are currently embedded in smart devices are widely used. It is prevailing to utilize wearables which have embedded sensors to collect data, such as smartphones [7], due to the reason that wearable devices are convenient to carry and suitable for long-term monitoring.

The wearable sensors that are used to collect raw data are varied [8,9,10,11]. For example, there are accelerometer, gyroscope, magnetometer, barometer, light, proximity, GPS, and so on. Paraschiakos et al. focused on the task of activity recognition from accelerometer data by combining ankle and wrist accelerometers [12]. Elsts et al. also used wearable accelerometers to propose an energy-efficient activity recognition framework [13]. Additionally, Lawal et al. utilized accelerometer and gyroscope signals from seven different body parts to generate frequency image sequences for predicting human activity [14]. The accelerometer measures acceleration of subject in experiment to analyze human motion states. The gyroscope is commonly used to measure the angle of rotation and its rate of change. Thus, the combination of the two sensors is able to collect informative data, which include more representative features.

The activity recognition is to identify various types of human daily activities that are grouped according to different points of view. For example, the main monitoring activities are classified into static activity, dynamic activity [15] and transitional activity according to activities’ characteristics, which is also studied in this paper. Generally, the static activities (e.g., standing, sleeping) and the dynamic activities (e.g., walking, running) are focused in current researches, which have achieved significant performance. But the transitional activities (e.g., stand-to-sit, sit-to-stand) are more complicated due to their short duration, which cause easy loss of performance [16]. Although transitional activities have some overlaps with static and dynamic activities, it is indispensable to detect transitional activities in real life. A variety of activities are usually performed by people in a period of time, containing various transitional activities that include the end of an activity and the beginning of next activity. The transitional activity can also play a huge role in practical application. For patients, monitoring the transitional period makes great sense, which can help diagnosing patient’s physical state in real-time [17]. Nowadays, there is already clinical application that using Sit-to-Stand Test to judge safety and reliability with older intensive care unit patients at discharge [18]. Thus, transitional activities recognition is worth studying. Meanwhile, transitional activities are intermediate and short-term transitions between two activities, thus the number of instances of transitional activities is usually far less than static and dynamic activities in an experiment, which results in drops of accuracy due to the imbalance of sample number. In order to alleviate the sample imbalance problem, some methods can play an important role in it. Research has investigated this problem by improving the performance of the recognition model [19]. In addition to modifying model, it can also be alleviated from the perspective of data processing. Therefore, the resampling technique [20] is also used to improve unbalanced problem.

The activity recognition is often conducted by many conventional machine learning algorithms, such as KNN [21,22], SVM [23], Random Forest (RF) [24], and K-means [25]. The universal procedures which perform activity recognition by applying machine learning algorithms contain data gathering, data preprocessing, data segmentation, feature extraction, classifier training, and testing. Feature extraction is the core of procedure, which has a significant influence on classification performance. In this step, features are often extracted manually. Even though conducting activity recognition by applying machine learning algorithms have reached reasonable performance, they need high time-cost and computing resource for extracting features manually. Thus, deep learning methods that are able to extract features automatically have received more widespread attention on human activity recognition [26,27].

In this paper, the stacked denoising autoencoder (SDAE) [8], which is a kind of deep learning model utilized to perform activity recognition on account of its advantages. Firstly, it is able to extract features automatically comparing with conventional machine learning methods. Secondly, it is able to reduce data dimension to simplify calculation while preserving information, thus providing better conditions for learning in the final training stage. Thirdly, it utilizes encoder to compress unnecessary data and uses decoder to reconstruct data, which is able to extract useful higher level of representations [28]. Moreover, our experimental data are collected by using wearable sensors that contain accelerometer and gyroscope. The data from the two sensors that are tri-axial are three-dimensional [29], which describes meaningful human movement tendency in realistic space. Additionally we would focus on not only the static and dynamic activities recognition, but also transitional activities recognition. Thus, our activities’ types in this study contain three static activities (standing, sleeping, and watching TV), three dynamic activities (walking, running, and sweeping) and six transitional activities (stand-to-sit, sit-to-stand, stand-to-walk, walk-to-stand, lie-to-sit, and sit-to-lie).

The main contributions in this study are described, as follows:

1. In this paper, besides static and dynamic activities recognition, we also focus on utilizing deep learning models to perform transitional activities recognition which are more difficult and complicated than other two cases.
2. We have improved the problem of unbalanced samples due to relatively short duration characteristic of transitional activity by applying the resampling methods and adopted varied resampling methods to search optimal scenario.
3. A novel framework based on stacked denoising autoencoder is utilized to recognize three types of activities, which has achieved significant performances and compared with other classical methods to verify the effectiveness of SDAE model on activity recognition, especially for transitional activities.

The rest of this paper is organized as follows: Section 2 introduces existing related works about activity recognition and deep learning methods. Section 3 describes the overall framework about architecture of stacked denoising autoencoder model. Section 4 designs the experimental procedures and proposes the methods of data preprocessing. The results of experiment are presented and analyzed. Finally, the conclusions are drawn in Section 6.

## 2. Related Work

In activity recognition, the widespread usage of portable and wearable smart devices, such as smartphones and smartwatches that are embedded with sensors, have enabled the human activity data to be gathered large-scale. Before smart devices became ubiquitous, human activity recognition was cumbersome by attaching multiple sensors to a user’s body [30]. However, with the proliferation of affordable smart devices, it has become easier to collect information about user activity through sensors embedded in the device. Various methods of detecting human activities using acceleration sensors of smartphones have been proposed [31]. In the current study, conventional machine learning methods and deep learning methods are widely used due to their different characteristics.

### 2.1. Conventional Machine Learning Methods

In this study, the data we used came from accelerometer and gyroscope, which included three types of activities (static, dynamic, and transitional activities). Transitional activity recognition has greater complexity owing to short duration. Jorge-L took feature vectors as input for activity prediction using an SVM and utilized a filtering module to deal with transitions and fluctuations on the SVM module output [32]. Machine learning methods are widely used to predict transitional activities, such as SVM, KNN, Random Forest, and so on. Junhuai Li proposed a method based on conventional machine learning algorithms, they first utilized K-means to distinguish between basic activities and transitional activities and then used Random Forest classifier to recognize the two types of activities accurately [33]. In this paper, we exploit a deep learning model, stacked denoising autoencoder, in order to recognize transitional activities. Feature extraction influences the algorithm performance, computation time and complexity [34]. Conventional machine learning classification methods are often used to complete tasks based on the extracted features. For instance, a paper exploited discrete cosine transform to extract effective features and used PCA to reduce the dimension of feature [35]. At last, they applied multi-class Support Vector Machine (SVM) to recognize human activity. Decision Tree (DT) classifier [36] has the preferable performance in recognizing daily activities. Deep neural language model is exploited for the discovery of interleaved and overlapping activities [37]. The model builds hierarchical activities and captures the inherent complexities in activity details. Backpropagation techniques was used to train feedforward neural for complex human activity recognition in a smart home environment [38]. Although the algorithm outperformed the Hidden Markov Model [39], DT, and SVM, it requires a combination of manual feature extraction to achieve high performance accuracy. However, for these conventional machine learning algorithms, there have been a drawback that they need to manually extract features. There is no doubt that extracting features manually increases the time consuming, energy and model building procedure. Thus, in this direction, the deep learning model that extracts features automatically has received more considerable attention.

### 2.2. Deep Learning Methods

With the emergence of deep learning and increased computation powers, deep learning methods are being widely adopted for automatic feature learning in diverse areas, like natural language processing, image classification, and speech recognition [40,41,42]. Recently, it has been used to extract features automatically from data collected by mobile and wearable sensors, and then classify human activity. In many researches, Convolutional Neural Network (CNN) [43,44,45] and Denoising Autoencoder [46], Restricted Boltzmann Machines [47] were used to extract features from data automatically. In this study, the stacked denoising autoencoder technique is utilized for activity recognition. Denoising autoencoder was proposed by P. Vincent et al. [48] and then A. Wang et al. [46] proposed the denoising autoencoder technique to learn underlying feature representation in sensor data and then integrate it with a classifier trained into single architecture to obtain powerful recognition model. Stacked autoencoder was introduced to analyze human activity from data of motion sensors, which has a high performance [49]. Therefore, autoencoder methods have demonstrated significant approaches for automatic feature representation to learn latent feature representation for human activity monitoring and detection approach. What is more, stacked denoising autoencoder was utilized to recognize eight activities, which achieved high accuracy [8]. However, it also only focused on static and dynamic activities not transitional activities. Generally, stacked autoencoder provides compact feature representation from continuous unlabelled sensor streams to achieve robust and seamless implementation of human activity recognition system [34]. In addition to the above mentioned, deep learning methods also include Recurrent Neural Network (RNN) [50,51], Long Short-Term Memory (LSTM) [52], Deep Belief Networks (DBN) [53], and so on. In Table 1, some references that utilized deep learning methods were listed. These deep learning models also have the advantage that extract features automatically.

It is obvious that these experiments that adopted deep learning models in Table 1 achieved acceptable accuracy. Thus, deep learning methods not only extract features automatically, but also improve the experiments’ performance. Moreover, when compared to these deep learning models, the SDAE model is able to improve effectively the problem of gradient disappearance and compress data by utilizing encoder, which extracts more representative features. Moreover, the SDAE model can reduce dimension of data to simplify calculation and improve operation efficiency. Thus, in this study, we would focus on stacked denoising autoencoders to extract feature and recognize human activity.

## 3. Materials and Methods

**Figure 1:**
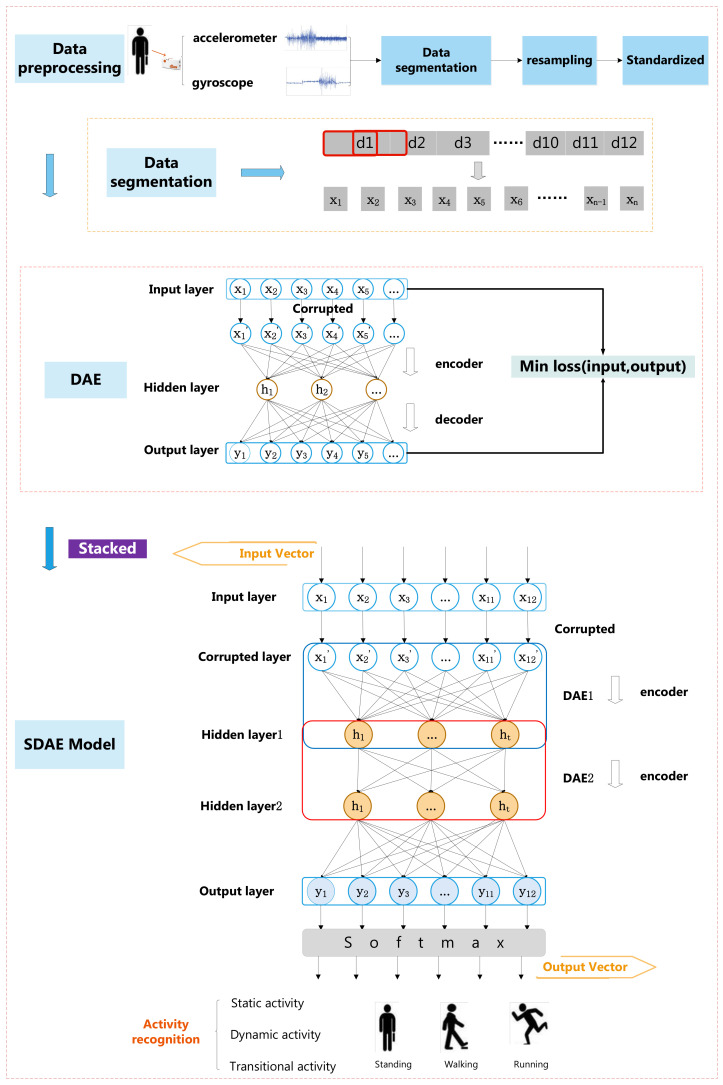
The overall framework of stacked denoising autoencoder (SDAE)-based activity recognition.

In this section, the overall framework of experiment and the method (SDAE) are described in detail, which are used to distinguish two types of activity on human activity recognition. The SDAE is a kind of deep learning model that extracts features automatically. The framework includes two parts: Data preprocessing and SDAE model, which is illustrated in Figure 1.

### 3.1. Data Preprocessing

After collecting data, data preprocessing is a critical procedure, which includes data segmentation, resampling and standardized, as is shown in Figure 1. In our experiment, the data are collected in a controlled lab environment by using wearable accelerometer and gyroscope. Ten healthy adults were chosen to perform twelve activities. At first, data segmentation was carried out in order to improve recognition accuracy. Then, owing to scarcity of transitional activity samples, the problem of unbalanced samples will drop recognition accuracy. The resampling technique was applied in order to improve this problem. Moreover, data standardization was also an important procedure to promote performance of experiment.

The dataset that we collected contains three primary types of activities: static activity, dynamic activity, and transitional activity. The static activity includes standing, sleeping, and watching TV. The dynamic activity includes walking, running, and sleeping. The transitional activity includes stand-to-sit, sit-to-stand, stand-to-walk, walk-to-stand, lie-to-sit, and sit-to-stand. All of the experimental data are collected by leveraging accelerometer and gyroscope, which have three-axis, $x_{i}=\left\{ac_{i}^{x},ac_{i}^{y},ac_{i}^{z},gy_{i}^{x},gy_{i}^{y},gy_{i}^{z}\right\}$. The preprocessing procedure is needed to be carried out before the experimental data become input of the model.

#### 3.1.1. Segmentation

Data segmentation is an essential procedure in human activity recognition, which is directly related to the recognition performance. In realistic world, human activities are continuous behaviors so that a single data sample at a time point cannot reflect enough tendency of an activity. Thus, it is necessary to segment data before training neural network. More representative features of each activity are extracted after data segmentation. In our experiment, the sliding window with 50% overlap is utilized to segment dataset, which has a significant influence on recognition performance. Thus, the dataset is segmented by integrating *n* samples as a sequence according to sampling rate, namely

(1) $$\begin{array}{c} x_{ac}=\left\{ac_{k}^{x},\ldots ,ac_{k+n-1}^{x},ac_{k}^{y},\ldots ,ac_{k+n-1}^{y},ac_{k}^{z},\ldots ,ac_{k+n-1}^{z}\right\}. \end{array}$$

(2) $$\begin{array}{c} x_{gy}=\left\{gy_{k}^{x},\ldots ,gy_{k+n-1}^{x},gy_{k}^{y},\ldots ,gy_{k+n-1}^{y},gy_{k}^{z},\ldots ,gy_{k+n-1}^{z}\right\}. \end{array}$$

(3) $$x_{i}=\left\{x_{ac},x_{gy}\right\}.$$

where $x_{i}$ is a 1×M vector (*M* equals to 3072 in this study), $x_{ac}$ represents raw data from accelerometer, $x_{gy}$ represents raw data from gyroscope, and *k* is sequence size. The *n* is set to 512 in this paper according to sample rate of 102.4 Hz and time interval of five seconds, which is used to combine 512 samples into one instance. A sliding window whose size is 512 with a 50% overlap is utilized to complete this process. After segmenting, every instance corresponds to a specific activity class. By this way, we acquired more powerful performance of classifier.

#### 3.1.2. Resampling

After segmentation, we then choose whether to apply resampling technique or not according to the distribution of samples. That is because the accuracy of model drops when the distribution of samples is unbalanced. In general, in order to improve the unbalanced samples problem, the common used method is the resampling technique, such as random undersampling, random oversampling and Synthetic Minority Oversampling Technique (SMOTE) [54,55,56].

Random undersampling is able to promote running efficiency by decreasing the number of training dataset when the dataset is too big, but it may discard some important information, which results in inaccuracy of model. Random oversampling is able to duplicate some classes, which just has few samples to increase the number of minority class and make sample size become balanced, but it may cause overfitting problem due to random duplicate. Despite all of this, the SDAE model that we utilized is able to decrease noise and, therefore, mitigate overfitting problem. The SMOTE method analyzes samples of minority class and synthesize new instances, and then adds these synthetic samples to dataset. More specifically, for each sample in minority classes, their near neighbors can be acquired according to euclidean distance between them and other samples. Subsequently, some samples are chosen randomly to create new samples by applying Equation (4). These examples would be added to the original dataset.

(4) $$x_{new}=x+rand\left(0,1\right)*|x-x_{n}|.$$

where $x_{n}$ is a sample in minority class and *x* is a random nearest neighbor sample of $x_{n}$. rand(0,1) represents a random number between 0 and 1, $|x-x_{n}|$ calculate euclidean distance between *x* and $x_{n}$. By this way, it alleviates overfitting problem effectively and does not discard meaningful information. However, the SMOTE also has its faults, it may generate extra noise when creating new instances.

These methods are able to improve unbalanced samples problem and they have specific drawbacks. We would discuss and analyze their performance in next section. Moreover, data standardization was carried out before it was taken as input of model in order to improve the accuracy. In this step, each value is mapped to the 0–1 range by applying mean and standard deviation:

(5) $$x^{*}=\frac{x-\mu }{\sigma }.$$

where $x^{*}$ represents the input data, *x* represents concrete value, μ is mean and σ is standard deviation. In the end, the whole dataset was divided into training set, validation set, and testing set with the ratio of 6:2:2 randomly.

### 3.2. Stacked Denoising Autoencoder

The stacked denoising autoencoder stacks input layers and hidden layers of multiple denoising autoencoders (DAE). Figure 1 illustrates its architecture and overall design in this paper. DAE includes an input layer, a hidden layer, and an output layer, which utilizes encoder and decoder to acquire output. The encoder can encode input data to learn hidden features. Subsequently, the decoder is able to decode the output of encoder to restructure data. Its goal is to minimize the loss between input and output and make them keep consistent as far as possible. What is more, a denoising factor is used to corrupt input in order to avoid the problem that output is a direct copy of raw input. The training process of stacked denoising autoencoder consists two steps: (i) pretraining; (ii) fine-tuning.

#### 3.2.1. Pretraining

In this step, the stacked denoising autoencoder utilizes greedy layer-wise training to update parameters. In other words, every denoising autoencoder in stacked denoising autoencoder are trained separately. Moreover, every DAE’s output will act as input of subsequent denoising autoencoder until the whole network is trained.

There are two processes in training process of denoising autoencoder: encoding and decoding. In the encoding process, our training data from accelerometer and gyroscope will be mapped to hidden layer by applying a sigmoid function, which is used to compress data, and it is then reconstructed by applying a sigmoid function in the decoding process. The error between the input data and the output data would be minimized by using gradient descent.

Formally, let $x_{i}$ represents the input data, which have already been preprocessed and segmented. $x_{i}$ is a 1*N vector. The N equals 3072 in this paper. The whole dataset is represented, as follows:

(6) $$S_{n}=\left\{x_{0},x_{1},\ldots ,x_{n}\right\}.$$

Firstly, each $x_{i}$ must be corrupted by a denoising factor *a*, which obtain $x_{i}^{{}^{\prime }}$. The probability of each node lost in the layer is *a*. Subsequently, in the encoding section, $x_{i}^{{}^{\prime }}$ is mapped to hidden layer by a sigmoid function *f*, namely

(7) $$y=f(W_{1}x_{i}^{{}^{\prime }}+b_{1}).$$

where $W_{1}$ and $b_{1}$ are the weight matrix and bias, respectively. Afterwards, the *y* is mapped to output layer by a sigmoid function *g*, namely

(8) $$z=g(W_{2}y+b_{2}).$$

where $W_{2}$ and $b_{2}$ are the weight matrix and bias respectively. Subsequently, the error between the output data and raw data is computed. The loss function we utilize is cross entropy, which is written as:

(9) $$L\left(x_{i},z\right)=-\sum_{i=0}^{n}\left(x_{i}log\left(z\right)+\left(1-x_{i}\right)log\left(1-z\right)\right).$$

Last, the parameters of each layer are updated by applying gradient descent. After that, the pretraining process is done.

#### 3.2.2. Fine-Tuning

In this step, a softmax layer is added at the top of network, which is used to identify current type of activity. Afterwards, the whole network would be trained liked a Multilayer Perceptron (MLP) in a supervised manner using labeled data. It is noted that the parameters of pretraining progress are shared with fine-tuning progress. Subsequently, parameters of each layer are fine-tuned by applying backpropagation and gradient descent. In training progress, the whole dataset is divided into training set, validation set, and test set in proportion. The training set is used to train model, validation set is used to detect optimal model specifications, and test set is used to test the performance of model. The pseudo-code of human daily activity recognition in this paper is described in Algorithm 1.

**Algorithm 1** Human activity recognition method with SDAE.**Input:** Raw dataset *D***Output:** Activity types of testing dataset 1: Data Preprocessing: 2: Segment the dataset according to sampling frequency 3: Apply the random oversampling 4: Standardize the dataset to obtain input vector $x_{i}$ 5: Divide the dataset into training dataset $D_{train}$, validation dataset $D_{validate}$, test dataset $D_{test}$ 6: Pretraining: 7: **while**
l<= Hidden layers $N_{l}$
**do** 8:  The dataset $x_{i}$ in $D_{train}$ is corrupted into $x_{i}^{{}^{\prime }}$ by adding a denoising factor. Then let $x_{i}^{{}^{\prime }}$ as input to train l-th layer of stacked denoising autoencoder. 9:  The output of l-th layer will be the input of l + 1-th layer 10:  l += 1 11: **end while** 12: Fine-tuning:
 13: Fine-tune the whole network by applying backpropagation. Utilize labeled dataset $D_{train}$ to train softmax layer. 14: Test: 15: Use the $D_{train}$ and $D_{validate}$ to train model and validate performance of model respectively.  Recognize the activity type of test data $D_{test}$.

### 3.3. Experimental Design

This experiment was carried out in a controlled laboratory environment of the Ulster University and approved by an independent research/ethics review board in order to acquire efficient and useful information of activities. Ten healthy adults who came from the Ulster University were chosen to perform the twelve activities that were grouped into three classes: static activities, dynamic activities, and transitional activities. These activities contain standing, sleeping, watching TV, walking, running, sweeping, stand-to-sit, sit-to-stand, stand-to-walk, walk-to-stand, lie-to-sit, and sit-to-lie. Table 2 provides their brief descriptions.

In designing progress, every subject was attached a tri-axial accelerometer and a tri-axial gyroscope on their left wrist which is one of the most common placements in activity detection. Then they performed twelve activities according to the description of Table 2 by following a verbal guided sequence. Each activity will be performed in the predefined order above a rest period of one minute will be observed between each activity and each repeat. The subjects will carry out each activity five times to ensure the repeatability and reliability of measurement. Moreover, the researcher informed subjects what activity to perform, but not limit strictly how to perform. The raw data were collected by a Shimmer wireless sensing platform that contains an on-board tri-axial accelerometer and gyroscope with a configurable sampling rate up to 1 kHz and an amplitude range up to ±6 g. The Shimmer is a compact, wearable sensing platform, which runs on the TinyOS operating system. Sensor data from the SHIMMER are transmitted via Bluetooth to a PC. In reality, our original data were collected with a sampling rate of 102.4 Hz and an amplitude range of ±2.0 g (g = 9.8 m/s${}^{2}$), which was conducted by accelerometer and gyroscope sensors. Therefore, 4,020,288 samples were recorded over ten hours in the end.

## 4. Results

In this section, we will analyze the experimental results. After data preprocessing, the segmented dataset is taken as input of model to train. Meanwhile, various experimental methods will be used. In this step, we will discuss results in terms of different perspectives.

### 4.1. Experimental Result Without Resampling

In our study, there are 12 activities, which contain standing, sleeping, watching TV, walking, running, sweeping, stand-to-sit, sit-to-stand, stand-to-walk, walk-to-stand, lie-to-sit, and sit-to-lie. The recognition accuracy of every activity is shown in Table 3 in order to analyze the performance of experiment without resampling.

In experiment, the estimation metrics this paper adopted are Accuracy, Precision, Recall, and F1 score. The Accuracy is the proportion between number of true labels model predicted (the sum of true positives and true negatives) and all true labels (the sum of true positives, true negatives, false positives, and false negatives) (Equation (10)). The Precision of an activity is proportion between the number of correct predictions (true positives) and the number of corresponding activity’s label model predicted (the sum of true positives and false positives) (Equation (11)). The Recall of an activity is the proportion between the number of true label model predicted (true positives) and the number of this activity’s realistic label (the sum of true positives and false positives) (Equation (12)). The F1 score is the combination of the Precision and the Recall (Equation (13)), which can be defined, as follows:

(10) $$Accuracy=\frac{TP+TN}{TP+TN+FP+FN}$$

(11) $$\;Precision=\frac{TP}{TP+FP}$$

(12) $$Recall=\frac{TP}{TP+FN}$$

(13) $$F1\;score=2\times \frac{Precision\times Recall}{Precision+Recall}$$

where TP is true positive, TN is true negative, FP is false positive, and FN is false negative.

Table 3 shows the performance of static and dynamic activity recognition. It is obvious that they have achieved high accuracy and static activity recognition performance is better. However, the performance of transitional activities recognition is much lower than static and dynamic activities. For example, the walk-to-stand activity recognition obtained a worst accuracy of 26.52% which is bolded and the sleeping activity recognition achieved the best precision of 98.37%, which is bolded. The main reason for this result is that our dataset is unbalanced. The maximum gap has been up to 246,074 samples between transitional and other activities. The relatively short duration characteristic of transitional activities make the number of transitional activities be far less than static and dynamic activities. Meanwhile, it can be concluded that the problem of unbalanced samples will drop accuracy apparently.

Therefore, the resampling techniques are utilized to improve the problem of unbalanced samples in order to promote the performance of experiment.

### 4.2. Performance Enhancement with Resampling

**Figure 2:**
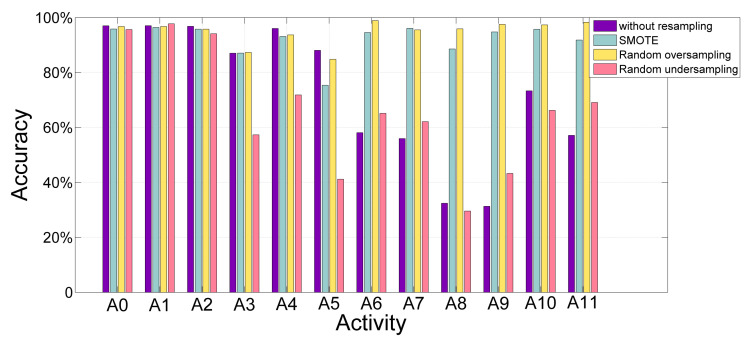
Accuracy of each activity class by applying random oversampling, SMOTE, and without resampling. (A0-standing, A1-sleeping, A2-watching TV, A3-walking, A4-running, A5-sweeping, A6-stand-to-sit, A7-sit-to-stand, A8-stand-to-walk, A9-walk-to-stand, A10-lie-to-sit, and A11-sit-to-lie).

The primary reason of poor performance is that the distribution of samples in our study is extremely unbalanced. When compared to static and dynamic activities, the number of transitional activities is too little. As mentioned above, the maximum gap has been up to 246,074 samples between transitional activity and dynamic activity, which decreases the accuracy obviously. Thus, the resampling technique is proposed in order to avoid the drops of accuracy. The random oversampling, SMOTE and random undersampling are applied to enhance performance. Table 4 shows the sample size of raw data and the instance size after segmentation and applying resampling technique. Obviously, the resampling methods have made the size of each class be same by increasing or decreasing samples. Figure 2 demonstrates the final performance of model by applying resampling technology.

According to Table 4, the sample size of different activities have large gaps and three resampling techniques have improved the imbalance sample problem. In Figure 2, it is obvious that performance of recognition has significant improvements after applying random oversampling and SMOTE. However, the random undersampling has a negative impact on the recognition accuracy, which may be due to the discard of too much useful information. Moreover, the random oversampling technique in this study has more powerful influence. Thus, it may be known, the problem of unbalanced samples does have significant influence on the results. When sample size is balanced, the accuracy has been greatly improved. Furthermore, it is noted that dynamic activities recognition performance has a slight drop. That is because dynamic activities are more complex than other activities and more likely to be misrecognized.

After resampling, the final classification performance of method in this study reached 94.88% as measured by Accuracy, 94.88% as measured by Precision, 94.88% as measured by Recall, and 94.86% as measured by F1 score. Meanwhile, the classification performance of each activities is shown in Table 5.

From Table 5, the SDAE model acquires a significant performance and the recognition of every activity contributes high accuracy. The balanced samples have played a great role in recognition performance. And transitional activity recognition has achieved high accuracy by utilizing the SDAE model. Furthermore, it is obvious that the performance of transitional activities precedes dynamic activities. The sweeping and walking reach lowest accuracy than other activities. We assume that, when compared to dynamic activity, the transitional activity has more obvious changes in a short time, which make the activities’ features more representative. According to bolded results, the sweeping achieves a lowest accuracy of 84.81%, the stand-to-sit reaches a highest accuracy of 98.92%.

**Figure 3:**
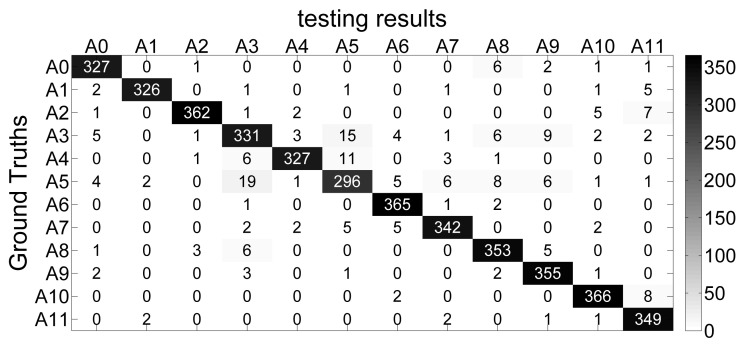
The confusion matrix obtained by applying random oversampling.

A confusion matrix is given, the most obvious errors are that 15 instances of walking are incorrectly recognized as sweeping, and 19 instances of sweeping are misrecognized as walking, as is shown in Figure 3. It is concluded that walking activity and sweeping activity have similar characteristics to some extent. Thus, the two activities are easily misrecognized and reach lowest accuracy of recognition.

### 4.3. Hyperparameter Analysis

When adopted different hyperparameters, the classifier had different performance. Therefore, some specific parameters were configured in experiment to obtain the best performance. These parameters we discussed contains iterations of training progress, pretraining learning rate, fine-tuning learning rate and the number of hidden layer.

(a) Iterations of training progress

**Figure 4:**
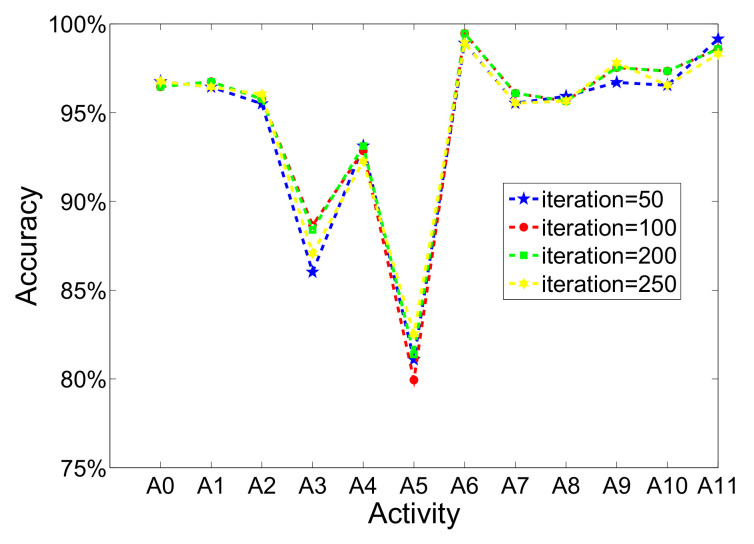
The recognition performance of each activity when using different iterations. (Pretraining learning rate is set to $1\times 10^{-7}$, number of hidden layer is set to 2; fine-tuning learning rate is set to 0.01).

In order to analyze effect of iterations, the accuracy changes of each activity are shown in Figure 4. With the increasing of iterations, the performance of each activity has no obvious changes. There are only three dynamic activities, walking, running, and sweeping, which have changed and have worse performance than static and transitional activities. However, it is noted that changes of different iterations are so small, which cannot transmit enough and useful information. Therefore, it is concluded that the number of iterations have not a significant influence on the performance of model. Moreover, when considering the accuracy, time-cost, and computational complexity, the choice of 200 iterations is a balance point. For each iteration, the training dataset is first used to train network, and then the loss of validation set and testing set can be obtained based on current parameters to detect recognition performance. This progress will repeat 200 times.

(b) Pretraining learning rate

**Figure 5:**
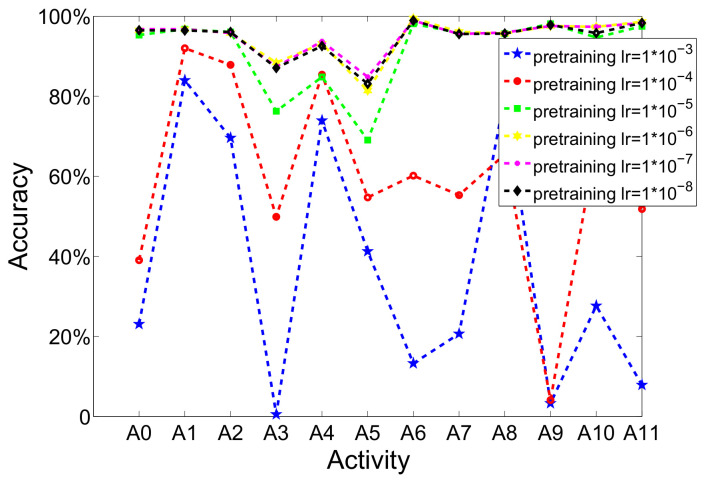
The recognition performance of each activity when using different pretraining learning rates (iterations is set to 200; number of hidden layer is set to 2; fine-tuning learning rate is set to 0.01).

Figure 5 indicates the influence of pretraining learning rate. When different pretraining learning rates are chosen, the performance had obvious changes. The performance of every activity was tested when the learning late is set to $1\times 10^{-3}$, $1\times 10^{-4}$, $1\times 10^{-5}$, $1\times 10^{-6}$, $1\times 10^{-7}$, $1\times 10^{-8}$, respectively, in order to analyze the influence of pretraining learning rate. As is shown in Figure 5, adopting different learning rates can lead to enormous fluctuations. Moreover, it demonstrates that the best performance is achieved at $1\times 10^{-7}$ level by comparing six parameters though the pretraining learning rate is set very low. Because the goal of pretraining is to acquire proper initial values of parameters and ensure that the fine-tuning process has better astringency and performance, the pretraining learning late does not need to be too high.

(c) Fine-tuning learning rate

**Figure 6:**
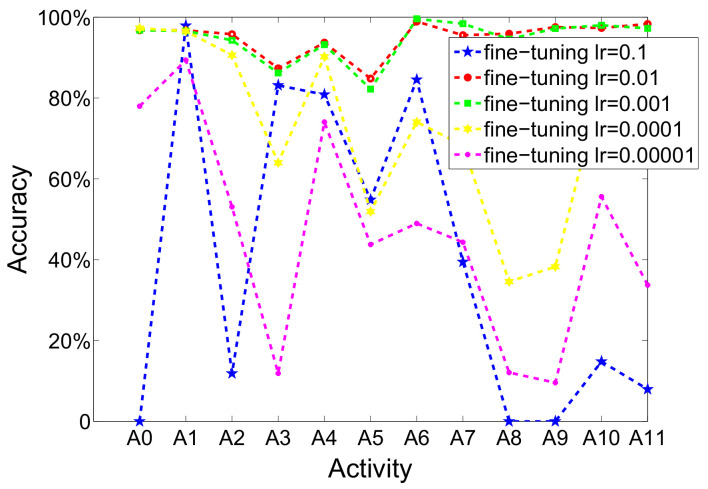
The recognition performance of each activity when using different fine-tuning learning rates (iterations is set to 200; number of hidden layer is set to 2; pretraining learning rate is set to $1\times 10^{-7}$).

In fine-tuning process of training model, backpropagation and gradient descent are used. The learning rate has played a significant role in this process. A set of experiments has been performed in order to search optimal setting of learning rate. Figure 6 shows the performance of activity recognition when adopting different fine-tuning learning rates. According to Figure 6, it is obvious that the accuracy will change evidently when the fine-tuning learning rate became smaller. It is different from pretraining learning rate, it does not need to be set too low. It will affect the convergence rate of model. When it is set to 0.1, 0.0001 or 0.00001, the recognition performance is very poor. Additionally, the recognition accuracy is significant when adopting 0.01 or 0.001, both of the two choice have similar results. Yet, when the learning rate is set to 0.1, the neural network can converge to global optimal solution quickly. Thus, we select 0.01 as fine-tuning learning rate of model.

(d) The number of hidden layer

**Figure 7:**
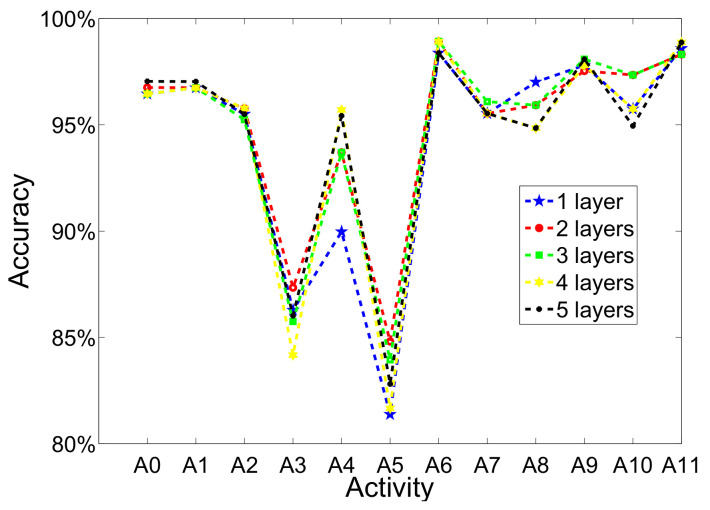
The performance of each activity recognition when selecting different numbers of hidden layer (iterations is set to 200, pretraining learning rate is set to $1\times 10^{-7}$; fine-tuning learning rate is set to 0.01).

In Figure 7, the performance fluctuations are exhibited when selecting different numbers of hidden layer. It is obvious that the main disparity concentrate on walking and sweeping. The both activities recognition achieve lowest accuracy than other activities, which is also as mentioned above. With the growth of hidden layer number, dynamic and transitional activities recognition performance are fluctuant while static activities are stable. Moreover, the fluctuation is not very obvious and computational cost will raise when using more layers. Thus, we utilize two hidden layers to create the network in order to achieve the trade-off between recognition accuracy and time-cost.

Subsequently, Table 6 has given a list of fixed hyperparameters of neural network architecture. Because some hyperparameters have little influence on recognition accuracy according to experiment, we have discussed the above four hyperparameters.

### 4.4. The Influence of Single and Several Sensors

**Figure 8:**
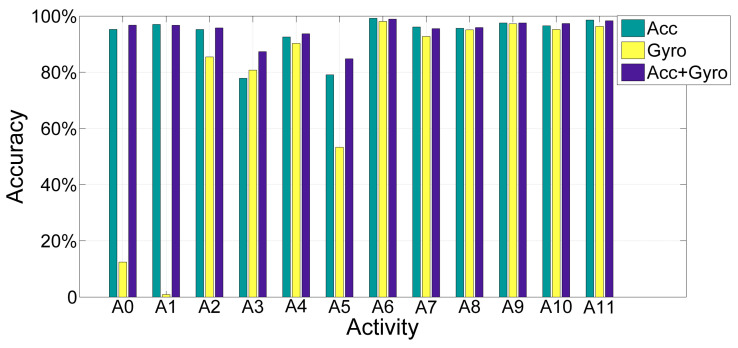
The performance changes of each activity recognition when adopting different sensors.

In this experiment, the data we collected were from accelerometer and gyroscope. In general, adopting more types of sensors has a stronger influence on results. To analyze how the two sensors affect the performance of model, Figure 8 is given to demonstrate the changes of specific activity when using different sensors. Table 7 shows the performance of accelerometer and gyroscope.

From Figure 8, the gyroscope improved indeed the accuracy of dynamic activities (walking, running, sweeping) recognition comparing to the single accelerometer. Moreover, it is obvious that, when exploiting the data collected by gyroscope identified the transitional activities, the performance of dynamically activity recognition is acceptable, but the static activity recognition is not efficient. When the data collected by accelerometer are used, the accuracy of static and transitional activity recognition was acceptable, but the dynamic activity was not recognized precisely. However, when the data collected by accelerometer and gyroscope together are used, all classes of activity recognition obtained a significant accuracy. Thus, utilizing two sensors has a good influence on result than one sensor.

According to the Table 7, although there is not much difference in the total performance of single accelerometer and two sensors, different activity recognition has different performance according to Figure 8. Specifically, a combination of accelerometer and gyroscope obtained a best performance. The single accelerometer reached an acceptable result and the single gyroscope achieved an unsatisfactory result. However, it is not able to conclude that the data from gyroscope are not worthy. In short, the combination of accelerometer and gyroscope contributes better performance than applying single sensor.

### 4.5. Comparison with Other Conventional Methods

In order to demonstrate the superiority of method of this paper, we compared it with some conventional machine learning algorithms, including Support Vector Machine (SVM), Decision Tree (DT), Naive Bayesian Model (NBM), K-nearest neighbors (KNN), and other deep learning algorithm, such as CNN, LSTM, and BiLSTM. In training progress of these conventional machine learning methods, the features we extracted are statistical features, including mean, maximum, minimum, variance, and standard deviation. Moreover, the random oversampling technique is also used to balance samples in these methods. By comparing with other methods, their performances are shown in Table 8.

From Table 8, it is obvious that the SDAE model obtains a best performance than other conventional machine learning algorithm (SVM, DT, KNN), which achieves accuracy of 94.88%. In conventional machine learning methods, SVM acquired the best performance that recognition accuracy exceeded 90% and other methods all have more than 80% of accuracy, which are lower than SDAE model. In experimental progress, we found that SDAE model needs longer runtime than conventional machine learning algorithms, but these methods need extra times to perform feature extraction and selection. SADE is able to extract features automatically, which can save energy. Thus, in activity recognition, it is more precise and convenient to utilize SDAE than conventional machine learning algorithms. Furthermore, compared to CNN and LSTM, the SDAE model acquires a more accurate result. Generally speaking, CNN and LSTM model can also achieve significant performance. In this situation, we analyze the accuracy of each activity after utilizing LSTM and CNN. We find that the watching TV has achieved the lowest accuracy of 42.39% than other activities that have achieved the accuracy of 76.02–95.87% when the LSTM model is used. The CNN model has faced a similar situation that the recognition accuracy of watching TV is the lowest. Thus, we assume that these models can acquire significant performance in some activities (e.g., running), but they cannot extract useful representation in some activities (e.g., watching TV), which have more similarities with other activities. We also use BiLSTM to recognize activities. It has a better performance than CNN and LSTM, but it also has a lower accuracy than the SDAE model. Additionally, we also find that they need more time to train than SDAE model. In point, the SDAE model is proper.

### 4.6. The Performance of SDAE Model on Three Public Datasets

With the experimental verification, the behavioral dataset we collected reach a significant performance on activity recognition. However, considering a special situation that the result of experiment may depend on the dataset, and not on the model. Therefore, without loss of generality, we also conducted different experiments that exploited three public datasets, which include human activity recognition using smartphones data set [57], activity recognition from single chest-mounted accelerometer data set [58], and UCI daily and sports dataset [59]. Table 9 shows their basic information and their performance in experiments are given in Table 10.

The SDAE model obtained significant performances on three public datasets, according to the Table 10. Thus, we are able to conclude that the SDAE model has strong applicability and the result of our experiment is convincing.

## 5. Discussion

The preceding results show the significant performance of SDAE model on transitional activities and illustrate that the accuracy will drop when dataset is unbalanced. The resampling technique provides feasible methods to improve this question and the accuracy can be greatly improved. In the experiment, we perform multiple sets of contrast experiments, which refer to resampling, hyperparameters, different sensors, multiple methods, and datasets. According to the resampling experimental result, the resampling technique is able to balance sample gap and enhance the recognition performance obviously. Additionally, the SDAE model can alleviate the overfitting problem due to its denoising characteristic. Different sensors have unique effect on activity recognition, the integration of varied wearable sensors can have a positive influence on performance. Additionally, it must be noted that the measurement accuracy of wearable sensors may have an effect on recognition performance. In this study, we have utilized a kind of accelerometer and a kind of gyroscope to collect data. Thus, we do not discuss it in detail and it will be considered in our future work. Moreover, we have analyzed the reason that CNN and LSTM achieve lower performance than SDAE. We assume that these models cannot apply to all human daily activities and the architecture need to be adjusted according to demand. Furthermore, the results on three public datasets based SDAE demonstrate the effectiveness and applicability of our framework. According to these experimental results, it can be concluded that SDAE is proper to recognize three types of activities in this paper, especially for transitional activities.

In activity recognition area, the transitional activity is as important as other activities and can be applied to healthcare filed. For example, it can play a significant role in human fall detection technique, detecting patient state, and so on. We believe there will be more research results about transitional activities in different fields in the future.

## 6. Conclusions

In this paper, we utilized the stacked denoising autoencoder, a deep learning model that is able to extract useful features automatically and compress unnecessary data by encoder to extract more representative features and recognize human daily activities. Moreover, besides static and dynamic activities, we also focused on transitional activities recognition which were more difficult for recognizing. Furthermore, we utilized random oversampling technology to improve problem that samples were unbalanced due to the short-term characteristic of transitional activities. The experimental result we obtained demonstrated the SDAE model was able to achieve significant performance on transitional activities recognition and outperformed other conventional machine learning methods on human daily activity recognition. Meanwhile, we analyzed the influence of sensors respectively and verified the effectiveness of the SDAE model on three public datasets for activity recognition, which indirectly proved that the result we obtained in our dataset is convincing.

In the future, we consider studying further special human’s activity recognition, which will be able to be applied to specific fields, such as healthcare. Additionally, we also consider utilizing more kinds of sensors to collect data in future experiments. Furthermore, when considering various channels of data gathering, the combination of activity recognition and computer vision is also an important research.

## Figures and Tables

**Table 1:** References of applying deep learning methods on activity recognition (the Acc is accelerometer, the Gyr is gyroscope, the Mag is magnetometer, and the Bar is barometer).

Reference	Method	Sensor	Activity Classes	Accuracy
Gu et al. [8]	SDAE	Acc+ Gyr+ Mag+ Bar	stilling, running, walking, upstairs, downstairs, upElevator, downElevato, falsemotion	94.34%
Charissa et al. [43]	CNN+ tFFT	Acc+ Gyr	walking sitting, upstairs, downstairs, standing, laying	95.75%
Song-Mi et al. [44]	1D-CNN	Acc	run, walk, still	92.71%
Mario [45]	CNN	Acc	walking, sitting, jumping, lying, climbing_up, standing, running, climbing_down	94%
Masaya Inoue et al. [50]	RNN	Acc	standing, sitting, downstairs, laying, walking, upstairs	95.42%
Yao et al. [51]	CNN+ RNN	Acc+ Gyr	standing, climbStair-down, biking, walking, sitting, climbStair-up	94.20%
Yu et al. [52]	LSTM	Acc+ Gyr	walking, upstarirs, standing, sitting, downstairs, laying down	93.79%
Zhang et al. [53]	DBN	Acc	walking, running, standing, sitting, upstairs, downstairs, lying	98.60%

**Table 2:** The concrete descriptions of twelve human daily activities.

Class	Activities	Description
Stationary Activities	standing	The subject stands still and maintains 5 min
	sleeping	The subject sleeps on the sofa for 5 min and is allowed to do some small movements, such as changing the lying posture
	watching TV	The subject watches TV for 5 min when he sits on the sofa in a comfortable position. And changing sitting posture is allowed
Dynamic Activities	walking	The subject walks on treadmill at constant speed for 5 min
	running	The subject runs on treadmill for 5 min
	sweeping	The subject sweeps in room with vacuum cleaner for 5 min
Transitional Activities	stand-to-sit	Standing for 15 s, and then sitting on the sofa, repeat 15 times
	sit-to-stand	Sitting on the sofa for 10 s, and then standing up, repeat 15 times
	stand-to-walk	Standing for 15 s, and then walking for 15 s, repeat 15 times
	walk-to-stand	Walking for 15 s, and then standing for 15 s, repeat 15 times
	lie-to-sit	Sitting on the sofa for 15 s, and then lying down, repeat 15 times
	sit-to-lie	Lying on the sofa, and then sitting on the sofa, repeat 15 times

**Table 3:** Performance of each activity class recognition without resampling.

	Accuracy (%)	Precision (%)	Recall (%)	F1 Score (%)
standing	97.03	94.72	97.03	95.86
sleeping	97.32	**98.37**	97.32	97.84
watching TV	96.82	92.54	96.82	94.63
walking	86.75	85.71	86.75	86.25
running	95.73	91.81	95.73	93.73
sweeping	88.92	82.52	88.92	85.60
stand-to-sit	62.16	63.01	62.16	62.59
sit-to-stand	51.19	70.49	51.19	59.31
stand-to-walk	35.14	39.39	35.14	37.14
walk-to-stand	**26.51**	51.16	26.51	34.92
lie-to-sit	73.33	68.75	73.33	70.97
sit-to-lie	58.73	58.73	58.73	58.73

**Table 4:** The number of sample of raw data and the number of instance after segmentation and applying the resampling technique.

	Initial Number	After Segmentation	Undersampling	SMOTE	Oversampling
standing	307,061	1198	238	1200	1200
sleeping	307,109	1200	238	1200	1200
watching TV	306,228	1196	238	1200	1200
walking	300,457	1174	238	1200	1200
running	294,676	1151	238	1200	1200
sweeping	302,052	1179	238	1200	1200
stand-to-sit	61,173	239	238	1200	1200
sit-to-stand	61,035	238	238	1200	1200
stand-to-walk	61,881	242	238	1200	1200
walk-to-stand	61,640	242	238	1200	1200
lie-to-sit	61,454	240	238	1200	1200
sit-to-lie	62,089	242	238	1200	1200

**Table 5:** The classification performance of each activity by using random oversampling technique.

	Accuracy (%)	Precision (%)	Recall (%)	F1 Score (%)
standing	96.75	95.61	96.75	95.73
sleeping	96.74	98.79	96.74	97.75
watching TV	95.77	98.37	95.77	96.85
walking	87.34	89.46	87.34	88.39
running	93.70	97.61	93.70	95.62
sweeping	**84.81**	89.97	84.81	87.31
stand-to-sit	**98.92**	95.80	98.92	97.34
sit-to-stand	95.53	96.07	95.53	95.80
stand-to-walk	95.92	93.39	95.92	94.64
walk-to-stand	97.53	93.92	97.53	95.69
lie-to-sit	97.34	96.32	97.34	96.83
sit-to-lie	98.31	93.57	98.31	95.88

**Table 6:** Optimal hyperparameters for neural network.

Hyperparameter	Value
number of hidden layers	2
number of units per layer	500
pretraining learning rate	$1\times 10^{-7}$
fine-tuning learning rate	0.01
iteration	200
denoising factor	0.5
data segment size (in seconds)	5

**Table 7:** Performance comparison of adopting different sensors.

Sensors	Accuracy (%)	Precision (%)	Recall (%)	F1 Score (%)
Acc	93.36	93.35	93.36	93.27
Gyro	75.76	74.77	75.76	72.60
Acc+Gyro	94.88	94.88	94.88	94.86

**Table 8:** Performance comparison between SDAE and other methods.

Methods	Accuracy (%)	Precision (%)	Recall (%)	F1 Score (%)
SVM	90.95	90.81	90.95	90.60
DT	88.15	87.53	88.15	87.54
KNN	84.84	84.38	84.84	84.29
CNN	81.33	79.85	81.33	80.27
LSTM	81.63	83.56	81.63	81.62
BiLSTM	84.75	85.23	84.75	84.63
SDAE	94.88	94.88	94.88	94.86

**Table 9:** Basic information of the three public datasets.

Datasets	People	Classes	Sensors	Transitions
Smartphone	30	6	Acc+Gyro	No
Chest-mounted	15	7	Acc	No
UCI	8	19	Acc+Gyro+Mag	No

**Table 10:** Activity recognition performance of SDAE model on other public datasets.

Datasets	Accuracy(%)	Precision(%)	Recall(%)	F1 Score(%)
Smartphone	97.15	97.19	97.15	97.15
Chest-mounted	89.99	89.96	89.99	89.83
UCI	95.26	95.42	95.26	95.15
